# Profile and characteristics of violence against older adults during the COVID-19 pandemic

**DOI:** 10.1590/1518-8345.6220.3825

**Published:** 2023-01-30

**Authors:** Camila de Morais Ranzani, Sara Cirillo Silva, Paula Hino, Mônica Taminato, Meiry Fernanda Pinto Okuno, Hugo Fernandes

**Affiliations:** 1 Universidade Federal de São Paulo, Escola Paulista de Enfermagem, São Paulo, SP, Brazil.; 2 Scholarship holder at the Conselho Nacional de Desenvolvimento Científico e Tecnológico (CNPq), Brazil.

**Keywords:** Aged, Violence, Elder Abuse, Mandatory Reporting, COVID-19, Public Health Surveillance, Idoso, Violência, Abuso de Idosos, Notificação de Abuso, COVID-19, Vigilância em Saúde Pública, Anciano, Violencia, Abuso de los Ancianos, Notificación Obligatoria, COVID-19, Vigilancia de la Salud Pública

## Abstract

**Objective:**

to identify the sociodemographic profile and the characteristics of interpersonal violence against older adults during the first year of the COVID-19 pandemic in a capital city from the Brazilian Southeast region.

**Method:**

a descriptive and exploratory research study with a cross-sectional design based on the notifications of suspected or confirmed cases of violence against older adults between March 2020 and March 2021. A univariate statistical analysis and Fisher’s exact test (p<0.05) were performed.

**Results:**

a total of 2,681 notifications were recorded during the period. The main victims were individuals aged between 60 and 64 years old, female, white-skinned and with low schooling levels. The instances of violence were more frequent in the victims’ homes. Physical and psychological violence predominated, through physical force/beatings and threats, respectively. Most of the aggressors were male, younger than the victims and generally their children or intimate partners. The aggressions were perpetrated more than once and were driven by generational conflicts. There was low referral to entities for the protection of older adults.

**Conclusion:**

the sociodemographic profile found evidences vulnerable victims, subjected to many types of violence, and at a potential risk against their overall health.

## Resumo

**Objetivo:**

identificar o perfil sociodemográfico e as características da violência interpessoal contra a pessoa idosa no primeiro ano da pandemia COVID-19 em uma capital da região sudeste do Brasil.

**Método:**

pesquisa descritiva, exploratória, com delineamento transversal a partir da análise das notificações de casos suspeitos ou confirmados de violência contra a pessoa idosa, ocorridas entre março de 2020 e março de 2021. Foi realizada a análise estatística univariada e teste exato de Fisher (p<0,05).

**Resultados:**

houve 2681 notificações no período. As principais vítimas foram pessoas com idade entre 60 e 64 anos, do sexo feminino, brancas e com baixa escolaridade. As ocorrências tiveram maior frequência nos domicílios. As violências físicas e psicológicas foram as mais comuns, com uso de força física/espancamento e ameaça, respectivamente. O agressor era, em sua maioria, do sexo masculino, mais jovem do que a vítima, geralmente filho ou parceiro íntimo. As agressões ocorreram mais de uma vez e foram motivadas por conflitos geracionais. Houve baixo encaminhamento para órgãos de proteção a pessoa idosa.

**Conclusão:**

o perfil sociodemográfico encontrado evidencia vítimas vulneráveis, sujeitas a muitas formas de violência e com potenciais riscos à integralidade de sua saúde.

## Resumen

**Objetivo:**

identificar el perfil sociodemográfico y las características de la violencia interpersonal contra los adultos mayores en el primer año de la pandemia de COVID-19 en una ciudad capital de la región sureste de Brasil.

**Método:**

investigación descriptiva, exploratoria con diseño transversal a partir del análisis de las notificaciones de casos sospechosos o confirmados de violencia contra el adulto mayor, ocurridos entre marzo de 2020 y marzo de 2021. Se realizó un análisis estadístico univariado y la prueba exacta de Fisher (p< 0,05).

**Resultados:**

hubo 2681 notificaciones en el período. Las principales víctimas fueron personas entre 60 y 64 años, de sexo femenino, blancas y con baja escolaridad. La mayoría de los casos se registró en el hogar. La violencia física y psicológica fueron las más comunes, con uso de fuerza física/golpes y amenaza, respectivamente. El agresor era generalmente del sexo masculino, más joven que la víctima, hijo o pareja. Las agresiones se produjeron más de una vez y fueron motivadas por conflictos generacionales. Hubo baja derivación a organismos de protección de adultos mayores.

**Conclusión:**

el perfil sociodemográfico obtenido revela que son víctimas vulnerables, sujetas a múltiples formas de violencia y que la integridad de su salud está en riesgo potencial.

**Highlights**

(1) The main victims were women aged between 60 and 64 years old with low schooling levels.

(2) Physical and psychological aggressions were more frequent, with beatings and threats.

(3) The aggressors were mostly family members, men and younger than the victims.

(4) The aggressions were perpetrated more than once, driven by generational conflicts.

(5) Older adults were seldom referred to protection entities

## Introduction

Emergence of the new coronavirus disease (COVID-19) in 2018 imposed the need to adopt non-pharmacological measures to prevent and control it at the global level. Social distancing stands out among the measures, leading to the need for people to stay home with their families and with as little contact as possible with other individuals, favoring more opportunities for conflicts between family members, without any possibility of resolution in a short period of time and with high chances of violence^1^ ^-^ ^3^.

Environmental factors, stress and interpersonal relationship problems were intensified, reflecting on an increase in the number of domestic violence in several countries^4^. In February 2020, China reported a three-fold increase in the number of domestic violence incidents when compared to the previous year, with the #antidomesticviolenceduringepidemic hashtag resonating and being searched more than 3 million times on the social networks. In turn, France reported an increase from 32% to 36% in the number of cases, while in North America it varied between 21% and 35%^5^ ^-^ ^6^. The increase in the number of cases in Argentina and in the United Kingdom is estimated at 25%^6^. In April 2020, India recorded a 100% increase in the number of reports regarding this type of violence^7^. As attempts to mitigate this phenomenon, Italy and France decided to rent hotels to ensure protection of the victims, especially older adults^6^.

In Brazil, domestic violence during the COVID-19 pandemic is also a major concern. More vulnerable people, such as aged individuals, can become victims of physical, psychological and financial violence, among others types, and it is up to the health authorities and professionals to pay attention and search for dynamic and efficient coping measures to minimize or avoid such problem^8^ ^-^ ^9^.

Violence Against Older Adults (VAOA) represents a multicausal and complex process and is considered a serious public health problem associated with the individual and collective scopes. It is defined as actions, or as absence of appropriate actions, causing harm or anguish to older adults, as a result of using physical force, of sexual, psychological or financial aggression, or of neglect or abandonment^10^ ^-^ ^11^. In addition to these cases, institutional violence is also highlighted as a frequent manifestation in aged people living in Long-Term Institutions, which is equally difficult to identify and/or prevent.

A cohort study conducted with 897 older adults in the United States of America identified an 83.6% increase in older adults’ reports of abuse, when compared to the pre-pandemic period. It was estimated that one out of ten aged individuals in the United States of America had already been a victim of violence before adopting the social distancing measures. This number rose to one out of five older adults during the pandemic^11^. A scoping review that sought to map the available scientific evidence about VAOA during the COVID-19 pandemic identified that the publications still have a low level of evidence and that the gap regarding the theme hinders developing public policies to address this phenomenon^12^. Both research studies indicated that social protection measures should be planned in epidemic and pandemic situations to welcome the victims and avoid consequences to older adults’ overall health^11^ ^-^ ^12^.

Given the above, the authors formulated the following question: Which is the sociodemographic profile and the characteristics of interpersonal violence against older adults during the COVID-19 pandemic? The hypothesis presented is that more vulnerable aged people (longer-lived, black- or brown-skinned, with disabilities and living in poorer regions) have been more common victims, with frequent psychological and/or moral aggressions perpetrated by family members. It is believed that the knowledge obtained may foster planning of actions, programs to prevent and control VAOA, and public policies to protect this population segment.

The objective of this study was to identify the sociodemographic profile and the characteristics of interpersonal violence against older adults during the first year of the COVID-19 pandemic in a capital city from the Brazilian Southeast region.

## Method

### Study design

A descriptive and exploratory research study with a cross-sectional approach^13^, guided by the *Strengthening the Reporting of Observational Studies in Epidemiology* (STROBE) initiative^14^.

### Locus and data collection

The data were collected in the capital city of São Paulo. Data collection was conducted by means of the diverse information pointed out in the forms for the notification of suspected or confirmed cases of interpersonal violence of the Information System for Notifiable Diseases (SINAN, available from https://portalsinan.saude.gov.br/).

The information was stored in the aforementioned system by the notifying services, such as Epidemiological Surveillance, hospitals, outpatient services and other public services. After processing, the data were made available by the São Paulo Municipal Health Department in the TabNet tabulating program, created by the Informatics Department of the Unified Health System and made available on the Internet with unrestricted access.

The capital city of São Paulo was chosen for being the most populous Brazilian city and with significant social and cultural diversity. In addition to that, the notification data for this city are made available on TabNet for public consultation faster than in the national system, in view of the dimensions and varied characteristics of access to the Information System for Notifiable Diseases of the Brazilian states and municipalities. During the search, the national system had tabulated data until 2018, whereas that of the capital city of São Paulo already had 2021 data available.

### Study variables

The following variables of the victims were analyzed: age group, gender, race/skin color, schooling, Health Coordinating Area corresponding to the victim’s residence (health coordinating areas are administrative areas of the São Paulo Municipal Health Department, which may differ from geographical divisions from other public management sectors), month and year of occurrence, locus, marital status, sexual orientation, gender identity, and presence of disorders or disabilities and their type. In relation to the aggressor, the variables investigated were the following: number of people involved, relationship or degree of kinship with the victim, gender, life phase of the probable perpetrator, motivation, suspected alcohol consumption, means of aggression and whether the violence was repeated. Information was also collected about the referrals made by the professionals who assisted the victims. Denomination for the variables followed the same naming conventions of the individual notification form for suspected or confirmed cases of interpersonal violence from the Brazilian Ministry of Health.

### Selection criteria

The inclusion criteria corresponded to notifications of suspected or confirmed cases of interpersonal violence against people aged at least 60 years old and treated in public or private health units from the city of São Paulo (SP). Duplicate notifications corresponding to the same occurrence were excluded, analyzing the most complete forms in these cases. It is noted that it was not possible to segregate suspected from confirmed cases of interpersonal violence because the notification form was the same for both situations. Furthermore, the TabNet program does not allow such segregation.

### Period

The period delineated corresponded to that of the notifications of cases of violence made between March 2020 and March 2021. The authors chose 13 collection months for the following reasons: the World Health Organization issued the pandemic decree on March 11^th^, 2020, and the social distancing measures began to be adopted by the municipality of São Paulo a few days later, around day 16. The TabNet system does not allow searching by specific days, only by months. Thus, to avoid the risk of losing data from the last two weeks of March 2020, the authors decided to include it in the time frame. Data collection took place between December 2021 and February 2022. Excel 2007 was used for data tabulation. 

### Data treatment and analysis

The univariate statistical analysis was performed in the R software, version 4.0.2. As the dataset consists of categorical variables, a descriptive data analysis was performed based on the determination of absolute and percentage simple frequencies. The Chi-square test or Fisher’s exact test were performed to verify the associations between the type of violence and the other variables (violence driver, means of aggression, if sexual violence occurred, place of occurrence, gender of the aggressor, life cycle of the aggressor, referral, age group, race, schooling, marital status and disability). A 5% (α=0.05) significance level was considered.

### Ethical aspects

The research ethics principles were observed. As this study was collected in an unrestricted public domain database, there was no need for analysis by any Research Ethics Committee, in accordance with international standards and with Resolution No. 466/12 of the National Health Council.

## Results

During the period defined there were 2,681 notifications of violence against older adults in São Paulo, SP. Most of the sociodemographic variables were associated with the types of violence recorded. Only the “sexual orientation” (p=0.681) and “gender identity” (p=0.631) variables did not show significant statistical values, as shown in Table 1.

**Table 1:** Notifications of violence against older adults according to the victims’ sociodemographic characteristics. São Paulo, SP, Brazil, 2022

	Type of violence							
	Physical	Psychological/Moral	Torture	Sexual	Financial/ Economic	Neglect/ Abandonment	Legal intervention	*p*-value*
Variable	n (%)	n (%)	n (%)	n (%)	n (%)	n (%)	n (%)	
*Age group (years old)*								<0.001
60-64	526 (39.8)	198 (32.3)	11 (39.3)	29 (46.8)	31 (21.5)	70 (13.8)	3 (42.9)	
65-69	317 (24)	125 (20.4)	4 (14.3)	12 (19.4)	21 (14.6)	65 (12.8)	-	
70-74	189 (14.3)	105 (17.1)	7 (25)	10 (16.1)	32 (22.2)	87 (17.2)	-	
75+	288 (21.8)	185 (30.2)	6 (21.4)	11 (17.7)	60 (41.7)	285 (56.2)	4 (57.1)	
*Gender*								<0.001
Male	617 (46.7)	116 (18.9)	6 (21.4)	2 (3.2)	29 (20.1)	162 (32)	3 (42.9)	
Female	703 (53.3)	497 (81.1)	22 (78.6)	60 (96.8)	115 (79.9)	345 (68)	4 (57.1)	
*Race/Skin color*								0.004
Unknown/Blank	103 (7.8)	22 (3.6)	1 (3.6)	3 (4.8)	5 (3.5)	38 (7.5)	0 (0)	
White	617 (46.7)	327 (53.3)	19 (67.9)	28 (45.2)	83 (57.6)	273 (53.8)	4 (57.1)	
Black	154 (11.7)	71 (11.6)	1 (3.6)	7 (11.3)	18 (12.5)	66 (13)	0 (0)	
Asian	12 (0.9)	9 (1.5)	1 (3.6)	0 (0)	2 (1.4)	6 (1.2)	0 (0)	
Brown	430 (32.6)	182 (29.7)	6 (21.4)	23 (37.1)	35 (24.3)	124 (24.5)	3 (42.9)	
Indigenous	4 (0.3)	2 (0.3)	0 (0)	1 (1.6)	1 (0.7)	0 (0)	0 (0)	
*Schooling*								<0.001
Unknown/Blank	590 (44.7)	185 (30.2)	12 (42.9)	19 (30.6)	62 (43.1)	253 (49.9)	3 (42.9)	
Illiterate	37 (2.8)	24 (3.9)	1 (3.6)	5 (8.1)	11 (7.6)	29 (5.7)	0 (0)	
Incomplete 1^st^ to 4^th^ grade of Elementary School	210 (15.9)	130 (21.2)	7 (25)	10 (16.1)	30 (20.8)	105 (20.7)	0 (0)	
Complete 4^th^ grade of Elementary School	96 (7.3)	39 (6.4)	2 (7.1)	5 (8.1)	10 (6.9)	37 (7.3)	2 (28.6)	
Incomplete 5^th^ to 8^th^ grade of Elementary School	102 (7.7)	69 (11.3)	2 (7.1)	11 (17.7)	4 (2.8)	22 (4.3)	1 (14.3)	
Complete Elementary School	94 (7.1)	35 (5.7)	2 (7.1)	1 (1.6)	6 (4.2)	21 (4.1)	0 (0)	
Incomplete High School	39 (3)	24 (3.9)	1 (3.6)	3 (4.8)	2 (1.4)	9 (1.8)	0 (0)	
Complete High School	105 (8)	67 (10.9)	1 (3.6)	7 (11.3)	13 (9)	18 (3.6)	1 (14.3)	
Incomplete Higher Education	12 (0.9)	12 (2)	0 (0)	0 (0)	0 (0)	2 (0.4)	0 (0)	
Complete Higher Education	35 (2.7)	28 (4.6)	0 (0)	1 (1.6)	6 (4.2)	11 (2.2)	0 (0)	
*Health Coordinating Area (Residence)*								<0.001
West	72 (5.5)	24 (3.9)	0 (0)	0 (0)	2 (1.4)	10 (2)	0 (0)	
East	242 (18.3)	240 (39.2)	12 (42.9)	17 (27.4)	52 (36.1)	145 (28.6)	3 (42.9)	
North	218 (16.5)	78 (12.7)	3 (10.7)	12 (19.4)	17 (11.8)	87 (17.2)	1 (14.3)	
Southeast	221 (16.7)	73 (11.9)	2 (7.1)	5 (8.1)	18 (12.5)	60 (11.8)	0 (0)	
South	385 (29.2)	125 (20.4)	3 (10.7)	13 (21)	41 (28.5)	153 (30.2)	2 (28.6)	
Midwest	50 (3.8)	29 (4.7)	4 (14.3)	2 (3.2)	5 (3.5)	30 (5.9)	1 (14.3)	
Blank	132 (10)	44 (7.2)	4 (14.3)	13 (21)	9 (6.3)	22 (4.3)	0 (0)	
*Marital status*								<0.001
Blank/Unknown/ Not applicable	390 (29.5)	128 (20.9)	9 (32.1)	16 (25.8)	21 (14.6)	121 (23.9)	2 (28.6)	
Single	195 (14.8)	79 (12.9)	4 (14.3)	9 (14.5)	28 (19.4)	95 (18.7)	0 (0)	
Married/Consensual union	386 (29.2)	172 (28.1)	7 (25)	18 (29)	24 (16.7)	78 (15.4)	0 (0)	
Widowed	217 (16.4)	158 (25.8)	5 (17.9)	10 (16.1)	52 (36.1)	162 (32)	5 (71.4)	
Separated	132 (10)	76 (12.4)	3 (10.7)	9 (14.5)	19 (13.2)	51 (10.1)	0 (0)	
*Sexual orientation*								0.681
Heterosexual	857 (64.9)	399 (65.1)	15 (53.6)	42 (67.7)	92 (63.9)	309 (60.9)	5 (71.4)	
Homosexual	13 (1)	6 (1)	1 (3.6)	0 (0)	1 (0.7)	4 (0.8)	0 (0)	
Bisexual	2 (0.2)	1 (0.2)	0 (0)	0 (0)	0 (0)	0 (0)	0 (0)	
Does not apply	86 (6.5)	51 (8.3)	3 (10.7)	4 (6.5)	11 (7.6)	53 (10.5)	1 (14.3)	
Unknown	362 (27.4)	156 (25.4)	9 (32.1)	16 (25.8)	40 (27.8)	141 (27.8)	1 (14.3)	
*Gender identity*								0.631
Transvestite	4 (0.3)	0 (0)	0 (0)	0 (0)	0 (0)	1 (0.2)	0 (0)	
Transsexual woman	10 (0.8)	4 (0.7)	0 (0)	1 (1.6)	1 (0.7)	4 (0.8)	0 (0)	
Transsexual man	1 (0.1)	2 (0.3)	0 (0)	0 (0)	1 (0.7)	0 (0)	0 (0)	
Does not apply	829 (62.8)	410 (66.9)	18 (64.3)	41 (66.1)	99 (68.8)	342 (67.5)	4 (57.1)	
Unknown	476 (36.1)	197 (32.1)	10 (35.7)	20 (32.3)	43 (29.9)	160 (31.6)	3 (42.9)	
*Presence of disabilities*								<0.001
Blank/Unknown	244 (18.5)	116 (18.9)	2 (7.1)	13 (21)	22 (15.3)	75 (14.8)	2 (28.6)	
Yes	347 (26.3)	95 (15.5)	5 (17.9)	11 (17.7)	41 (28.5)	193 (38.1)	1 (14.3)	
No	729 (55.2)	402 (65.6)	21 (75)	38 (61.3)	81 (56.3)	239 (47.1)	4 (57.1)	
*Type of disability/disorder*								<0.001
Physical disability	42 (3.2)	30 (4.9)	1 (3.6)	3 (4.8)	12 (8.3)	79 (15.6)	0 (0)	
Intellectual disability	22 (1.7)	8 (1.3)	0 (0)	1 (1.6)	2 (1.4)	34 (6.7)	0 (0)	
Visual disability	15 (1.1)	15 (2.4)	1 (3.6)	0 (0)	5 (3.5)	41 (8.1)	1 (14.3)	
Auditory disability	12 (0.9)	10 (1.6)	0 (0)	0 (0)	7 (4.9)	26 (5.1)	0 (0)	
Mental disorder	76 (5.8)	27 (4.4)	3 (10.7)	6 (9.7)	13 (9)	34 (6.7)	0 (0)	
Behavioral disorder	20 (1.5)	10 (1.6)	2 (7.1)	0 (0)	3 (2.1)	25 (4.9)	0 (0)	
No information	1,133 (85.8)	513 (83.7)	21 (75)	52 (83.9)	102 (70.8)	268 (52.9)	6 (85.7)	

*Fisher’s exact test.

It is noticed that physical violence was the most common type (49.2%; n=1,320), followed by psychological or moral (22.8%; n=613). People aged between 60 and 64 years old were the main victims of different types of violence, with the exception of situations of neglect or abandonment in individuals aged 75 and over (56.2%; n=285), as well as in financial or economic violence in the same age group (41.7%; n=60). The most abused gender was the female one in all types, with greater discrepancies between the genders in situations of sexual (96.8%; n=60) and psychological or moral violence (81.1%; n=497). Regarding race or skin color, all the manifestations of violence were more frequent among white-skinned people.

The victims’ schooling level stands out. A high number of cases with schooling level described as unknown or blank (41.9%; n=1,124) is evidenced in the frequency distribution. However, the second data highlighted is the number of aged people with incomplete schooling from 1^st^ to 4^th^ grade of Elementary School in almost all the violence variables (18.3%; n=492), with the exception of sexual violence, where 17.7% (n=11) of the older adults had finished 5^th^ to 8^th^ grade of the Elementary School. 

In relation to the health coordinating office where the cases of violence took place, it is evidenced that physical violence and neglect or abandonment were more common in the South region of the metropolis (20%; n=538), while the other manifestations occurred more frequently in the East region (12%; n=324). The main place of occurrence for all types of violence was the victims’ residence, with a higher relative frequency for financial/economic violence (91%; n=131). 

Older adults that were married or living in a consensual union showed higher prevalence values of psychological or moral violence (28.1%; n=172) and of sexual violence (29%; n=18). In turn, the situations of financial or economic violence were more frequently perpetrated against widowed individuals (36,1%; n=52), as well as the neglect or abandonment situations (32%; n=162).

It was evidenced that most of the older adults (56.4%; n=1,514) had no disabilities or disorders. When found, mental disorders were associated with physical violence (5.8%; n=76), whereas physical disability was associated with neglect and/or abandonment (15.6%; n=79).

The aggressors’ characteristics according to the type of event are presented in Table 2.

**Table 2:** Notifications of violence against older adults according to the aggressors’ characteristics. São Paulo, SP, Brazil, 2022

	Type of Violence							
	Physical	Psychological/Moral	Torture	Sexual	Financial/Economic	Neglect/Abandonment	Legal intervention	*p*-value*
Variable	n (%)	n (%)	n (%)	n (%)	n (%)	n (%)	n (%)	
*Gender of the probable aggressor*								<0.001
Male	831 (65.2)	383 (62.5)	21 (75)	57 (91.9)	65 (45.1)	157 (31)	4 (57.1)	
Female	283 (18.7)	157 (25.6)	4 (14.3)	2 (3.2)	51 (35.4)	144 (28.4)	0 (0)	
Both genders	51 (4)	59 (9.6)	1 (3.6)	1 (1.6)	25 (17.4)	135 (26.6)	3 (42.9)	
Unknown	155 (12.2)	14 (2.3)	2 (7.1)	2 (3.2)	3 (2.1)	71 (14)	0 (0)	
*Life phase of the probable aggressor*								<0.001
Child (0-9 years old)	9 (0.7)	6 (1)	0 (0)	1 (1.6)	1 (0.7)	1 (0.2)	0 (0)	
Adolescent (10-19 years old)	33 (2.5)	11 (1.8)	1 (3.6)	1 (1.6)	2 (1.4)	1 (0.2)	0 (0)	
Young person (20-24 years old)	90 (6.8)	28 (4.6)	2 (7.1)	1 (1.6)	8 (5.6)	15 (3)	1 (14.3)	
Adult person (25-59 years old)	695 (52.7)	402 (65.6)	20 (71.4)	33 (53.2)	94 (65.3)	293 (57.8)	4 (57.1)	
Older adult (60+ years old)	204 (15.5)	124 (20.2)	2 (7.1)	12 (19.4)	22 (15.3)	89 (17.6)	0 (0)	
Unknown	289 (21.9)	42 (6.9)	3 (10.7)	14 (22.6)	17 (11.8)	108 (21.3)	2 (28.6)	
*Suspected alcohol consumption*								<0.001
Yes	407 (30.8)	196 (32)	18 (64.3)	20 (32.3)	45 (31.3)	67 (13.2)	3 (42.9)	
No	462 (35)	325 (53)	5 (17.9)	23 (37.1)	59 (41)	278 (54.8)	3 (42.9)	
Unknown	447 (33.9)	89 (14.5)	5 (17.9)	19 (30.6)	40 (27.8)	160 (31.6)	1 (14.3)	
Blank	4 (0.3)	3 (0.5)	0 (0)	0 (0)	0 (0)	2 (0.4)	0 (0)	
*Relationship/Degree of kinship*								<0.001
Father	8 (0.6)	6 (0.9)	1 (3.3)	2 (3.2)	1 (0.6)	1 (0.2)	0 (0)	
Mother	7 (0.5)	14 (2.1)	0 (0)	0 (0)	2 (1.2)	5 (0.9)	0 (0)	
Stepfather	2 (0.2)	2 (0.3)	0 (0)	0 (0)	0 (0)	1 (0.2)	0 (0)	
Stepmother	2 (0.2)	1 (0.2)	1 (3.3)	0 (0)	1 (0.6)	0 (0)	0 (0)	
Spouse	165 (12.8)	117 (17.6)	4 (13.3)	14 (22.2)	11 (0.6)	24 (4.2)	0 (0)	
Former partner	40 (3.1)	23 (3.5)	0 (0)	8 (12.7)	7 (4.2)	9 (1.6)	0 (0)	
Boyfriend/Girlfriend	5 (0.4)	2 (0.3)	0 (0)	0 (0)	0 (0)	1 (0.2)	0 (0)	
Former boyfriend/girlfriend	3 (0.2)	4 (0.6)	0 (0)	1 (1.6)	0 (0)	0 (0)	0 (0)	
Son/Daughter	402 (31.2)	293 (44.1)	15 (50)	6 (9.5)	86 (51.5)	305 (53)	4 (50)	
Brother/Sister	42 (3.3)	24 (3.6)	0 (0)	1 (1.6)	8 (4.8)	44 (7.6)	0 (0)	
Friend/Acquaintance	144 (11.2)	40 (6)	2 (6.7)	12 (19)	10 (6)	23 (4)	0 (0)	
Unknown	215 (16.7)	20 (3)	2 (6.7)	14 (22.2)	5 (3)	5 (0.9)	0 (0)	
Caregiver	10 (0.8)	9 (1.4)	1 (3.3)	0 (0)	3 (1.8)	37 (6.4)	1 (12.5)	
Boss	2 (0.2)	1 (0.2)	0 (0)	1 (1.6)	1 (0.6)	2 (0.3)	0 (0)	
Person with institutional relationship	11 (0.9)	5 (0.8)	0 (0)	1 (1.6)	1 (0.6)	5 (0.9)	0 (0)	
Police officer/Law enforcement agent	5 (0.4)	0 (0)	0 (0)	0 (0)	0 (0)	1 (0.2)	2 (25)	
Self	57 (4.4)	11 (1.7)	1 (3.3)	1 (1.6)	2 (1.2)	24 (4.2)	1 (12.5)	
Others	169 (13.1)	92 (13.9)	3 (10)	2 (3.2)	29 (17.4)	89 (15.5)	0 (0)	
*Number of aggressors involved*								<0.001
One	933 (70.7)	440 (71.8)	20 (71.4)	52 (83.9)	86 (59.7)	221 (43.6)	2 (28.6)	
Two or more	253 (19.2)	163 (26.6)	8 (28.6)	8 (12.9)	53 (36.8)	229 (45.2)	5 (71.4)	
Unknown	127 (9.6)	7 (1.1)	0 (0)	2 (3.2)	5 (3.5)	54 (10.7)	0 (0)	
Blank	7 (0.5)	3 (0.5)	0 (0)	0 (0)	0 (0)	3 (0.6)	0 (0)	

*Fisher’s exact test.

It is evidenced that, in most types of violence, the number of people involved corresponded to a single aggressor (57.1%; n=1,531), with the exception of the neglect or abandonment cases, where there were two or more (45.2%; n=229). Most of the aggressors were the victims’ children, with cases where neglect and/or abandonment (53%; n=305) and physical violence (31.2%; n=402) were practiced standing out. In all the categories, male individuals (56.7%; n=1,518) aged between 25 and 59 years old (57.4%; n=1541) were indicated as probable aggressors. In relation to suspected alcohol consumption by the aggressor, no direct association was found with the types of violence. 

The “occurrence month” variable (p=0.497) did not show a significant statistical value. The main referrals made by the professionals who assisted the victims according to the types of violence were to services from the Health Network, as shown in Table 3.

**Table 3:** Notifications of violence against older adults according to the characteristics of the types of violence and to the referrals made. São Paulo, SP, Brazil, 2022

	Type of Violence							
	Physical	Psychological/ Moral	Torture	Sexual	Financial/ Economic	Neglect/Abandonment	Legal intervention	*p*-value*
Variable	n (%)	n (%)	n (%)	n (%)	n (%)	n (%)	n (%)	
*Occurrence month*								0.497
March 2020	109 (8.3)	40 (6.5)	1 (3.6)	8 (12.9)	9 (6.3)	38 (7.5)	0 (0)	
April 2020	65 (4.9)	23 (3.8)	0 (0)	2 (3.2)	4 (2.8)	16 (3.2)	0 (0)	
May 2020	82 (6.2)	35 (5.7)	2 (7.1)	2 (3.2)	8 (5.6)	45 (8.9)	0 (0)	
June 2020	79 (6)	42 (6.9)	1 (3.6)	4 (6.5)	12 (8.3)	37 (7.3)	0 (0)	
July 2020	115 (8.7)	59 (9.6)	3 (10.7)	4 (6.5)	17 (11.8)	49 (9.7)	0 (0)	
August 2020	127 (9.6)	58 (9.5)	4 (14.3)	2 (3.2)	8 (5.6)	46 (9.1)	1 (14.3)	
September 2020	100 (7.6)	58 (9.5)	4 (14.3)	4 (6.5)	15 (10.4)	46 (9.1)	0 (0)	
October 2020	117 (8.9)	46 (7.5)	2 (7.1)	2 (3.2)	12 (8.3)	35 (6.9)	1 (14.3)	
November 2020	99 (7.5)	50 (8.2)	4 (14.3)	7 (11.3)	9 (6.3)	41 (8.1)	2 (28.6)	
December 2020	116 (8.8)	54 (8.8)	5 (17.9)	7 (11.3)	18 (12.5)	44 (8.7)	0 (0)	
January 2021	99 (7.5)	43 (7)	1 (3.6)	11 (17.7)	8 (5.6)	26 (5.1)	1 (14.3)	
February 2021	115 (8.7)	56 (9.1)	1 (3.6)	4 (6.5)	10 (6.9)	50 (9.9)	2 (28.6)	
March 2021	97 (7.3)	49 (8)	0 (0)	5 (8.1)	14 (9.7)	34 (6.7)	0 (0)	
*Place of occurrence*								<0.001
Residence	875 (66.3)	557 (90.9)	24 (85.7)	48 (77.4)	131 (91)	454 (89.5)	5 (71.4)	
Collective housing	15 (1.1)	4 (0.7)	0 (0)	0 (0)	1 (0.7)	16 (3.2)	0 (0)	
Place of sports practice	3 (0.2)	0 (0)	0 (0)	0 (0)	0 (0)	0 (0)	0 (0)	
Bar or similar	16 (1.2)	0 (0)	0 (0)	0 (0)	1 (0.7)	1 (0.2)	0 (0)	
Street	185 (14)	15 (2.4)	2 (7.1)	4 (6.5)	0 (0)	5 (1)	1 (14.3)	
Shops/Services	17 (1.3)	5 (0.8)	0 (0)	1 (1.6)	1 (0.7)	3 (0.6)	1 (14.3)	
Industries/Construction	2 (0.2)	0 (0)	0 (0)	0 (0)	0 (0)	0 (0)	0 (0)	
Blank/Unknown	176 (13.3)	15 (2.4)	2 (7.1)	4 (6.5)	5 (3.5)	17 (3.4)	0 (0)	
Others	31 (2.3)	17 (2.8)	0 (0)	5 (8.1)	5 (3.5)	11 (2.2)	0 (0)	
*Means of aggression*								<0.001
Physical force/Beating	1,032 (63.5)	167 (30.2)	19 (33.9)	30 (42.9)	43 (32.1)	36 (14.3)	4 (57.1)	
Strangulation	48 (3)	20 (3.6)	6 (10.7)	3 (4.3)	6 (4.5)	2 (0.8)	0 (0)	
Blunt object	111 (6.8)	25 (4.5)	8 (14.3)	3 (4.3)	5 (3.7)	3 (1.2)	0 (0)	
Sharp object	97 (6)	19 (3.4)	5 (8.9)	1 (1.4)	6 (4.5)	2 (0.8)	0 (0)	
Hot substance/ object	9 (0.6)	2 (0.4)	3 (5.4)	1 (1.4)	1 (0.7)	0 (0)	0 (0)	
Poisoning/Intoxication	25 (1.5)	10 (1.8)	1 (1.8)	1 (1.4)	3 (35.1)	3 (1.2)	0 (0)	
Firearm	12 (0.7)	6 (1.1)	2 (3.6)	1 (1.4)	1 (0.7)	0 (0)	0 (0)	
Threat	216 (13.2)	212 (38.2)	12 (19.6)	26 (35.7)	47 (35.1)	43 (17.1)	2 (28.6)	
Other means	75 (4.7)	92 (16.8)	1 (1.8)	5 (7.1)	22 (16.4)	163 (64.7)	1 (14.3)	
*It occurred more than once*								<0.001
Blank	4 (0.3)	1 (0.2)	0 (0)	0 (0)	0 (0)	3 (0.6)	0 (0)	
Yes	562 (42.6)	481 (78.5)	22 (78.6)	32 (51.6)	114 (79.2)	301 (59.4)	5 (71.4)	
No	428 (32.4)	79 (12.9)	4 (14.3)	20 (32.3)	15 (10.4)	62 (12.2)	0 (0)	
Unknown	326 (24.7)	52 (8.5)	2 (7.1)	10 (16.1)	15 (10.4)	141 (27.8)	2 (28.6)	
*Violence driver*								<0.001
Sexism	64 (4.8)	42 (6.9)	2 (7.1)	22 (35.5)	8 (5.6)	6 (1.2)	0 (0)	
Homophobia/Biphobia/Transphobia	3 (0.2)	2 (0.3)	0 (0)	0 (0)	2 (1.4)	3 (0.6)	0 (0)	
Racism	1 (0.1)	1 (0.2)	0 (0)	0 (0)	0 (0)	0 (0)	0 (0)	
Religious intolerance	0 (0)	1 (0.2)	0 (0)	0 (0)	0 (0)	1 (0.2)	0 (0)	
Xenophobia	0 (0)	0 (0)	0 (0)	0 (0)	0 (0)	0 (0)	0 (0)	
Generational conflict	270 (20.5)	166 (27.1)	6 (21.4)	1 (1.6)	46 (31.9)	105 (20.7)	2 (28.6)	
Street situation	30 (2.3)	0 (0)	0 (0)	0 (0)	0 (0)	0 (0)	0 (0)	
Disability	10 (0.8)	13 (2.1)	1 (3.6)	0 (0)	4 (2.8)	16 (3.2)	0 (0)	
Others	337 (25.5)	122 (19.9)	3 (10.7)	11 (17.7)	30 (20.8)	148 (29.2)	3 (42.9)	
Does not apply	145 (11)	143 (23.3)	4 (14.3)	10 (16.1)	14 (9.7)	70 (13.8)	1 (14.3)	
Unknown	460 (34.8)	123 (20.1)	12 (42.9)	18 (29)	40 (27.8)	158 (31.2)	1 (14.3)	
*Referrals*								<0.001
Health Network (BHU, hospital)	917 (49.7)	418 (42.4)	19 (43.2)	39 (41.9)	106 (42.7)	406 (46.7)	4 (28.6)	
Social Assistance Network	181 (9.8)	190 (19.3)	7 (15.9)	17 (18.3)	65 (26.2)	256 (29.4)	2 (14.3)	
Education Network	2 (0.1)	3 (0.3)	0 (0)	0 (0)	1 (0.4)	3 (0.3)	0 (0)	
Women’s Care Network	91 (4.9)	128 (13)	2 (4.5)	23 (24.7)	7 (2.8)	2 (0.2)	1 (7.1)	
Children’s Protection Council	9 (0.5)	11 (1.1)	0 (0)	1 (1.1)	1 (0.4)	2 (0.2)	0 (0)	
Older Adults’ Protection Council	153 (8.3)	83 (8.4)	4 (9.1)	2 (2.2)	23 (9.3)	126 (14.5)	1 (7.1)	
Older Adults’ Service Police Station	103 (5.6)	48 (4.9)	3 (6.8)	1 (1.1)	18 (7.3)	39 (4.5)	0 (0)	
Reference Center in Human Rights	8 (0.4)	1 (0.1)	0 (0)	0 (0)	1 (0.4)	4 (0.5)	0 (0)	
Prosecutor’s Office	17 (0.9)	10 (1)	1 (2.3)	0 (0)	9 (3.6)	18 (2.1)	1 (7.1)	
Women’s Service Police Station	89 (4.7)	45 (4.6)	3 (6.8)	8 (8.6)	7 (2.8)	3 (0.3)	2 14.3	
Other Police stations	263 (14.3)	42 (4.3)	5 (11.4)	2 (2.2)	6 (2.4)	8 (0.9)	3 (21.4)	
Public Defense Office	11 (0.5)	8 (0.8)	0 (0)	0 (0)	4 (1.6)	3 (0.3)	0 (0)	

*Fisher’s exact test.

Physical force and/or beating was observed as the most common means of aggression in physical violence (63.5%; n=1,032). In turn, threats were the most frequent means in psychological/moral violence (38.2%; n=212). In all manifestations of violence against older adults, its occurrence was verified more than once (56.5%; n=1,517). In general, generational conflicts were found to be the drivers of the aggressions. However, the high number of “Others” and “Not applicable” answers stands out in all categories.

It is pointed out that the notification form for suspected or confirmed cases of interpersonal violence offers the possibility of indicating more than one referral for each case. The Health Network, which includes Basic Health Units (BHUs), general and specialized hospitals and outpatient services, among others, was the main referral destination, followed by the Social Assistance Network. The number of referrals to the Municipal Older Adults’ Protection Council was low when compared to other entities (p<0.001).

## Discussion

Unfortunately, the types of physical and psychological/moral violence against older adults are not uncommon. However, a number of studies carried out in Peru and Spain point out to an increase in these manifestations during the first year of the pandemic, when there were no vaccines or treatments and whose recommendations for non-pharmacological prevention included reducing movement of people and certain level of domestic confinement^1^ ^-^ ^2^. As a result, once lower, tensions were intensified and precipitated physical aggressions and offenses with powerful impacts on human rights and on older adults’ quality of life^4^.

VAOA does not usually affect a precise age group. In fact, depending on the driver, it is more associated with conditions of frailty and dependence than with age itself. However, a North American study mentions that the more advanced the age, the greater the aged person’s difficulty notifying the event, accessing services and seeking support networks^15^. It is common that, as in this study, younger aged individuals are the ones who most frequently report the maltreatment received, although this does not necessarily mean that they are the most victimized. National and international scholars^15^ ^-^ ^16^ mention that cases of neglect or abandonment and financial violence tend to be more frequent among longer-lived aged people because they are usually reported by others, such as neighbors, non-caregiver family members or friends. In addition to that, during consultations or appointments, young older adults may feel embarrassed to mention that they have been victims of financial violence or detail situations that they recognize as not very serious, when compared to physical and psychological violence. A similar fact can also occur in relation to race/skin color, where brown- and black-skinned people tend to naturalize offensive situations because they have already experienced them before, leading to underreporting^11^ ^,^ ^16^.

In this study, it was found that age people with low schooling levels are more frequent victims of all types of violence. Such evidence is corroborated by other studies^17^ ^-^ ^18^, indicating that it is also a reality in other countries around the world. However, a Brazilian study indicates that higher schooling levels are not necessarily a protective factor against violence^19^. In some cases, more educated people and also those with higher incomes feel embarrassed to report what happened or do not seek shelter services in situations of aggression^19^ ^-^ ^20^. However, the finding indicates that aged people with incomplete Elementary School deserve greater attention to the potential risk of violence. Another relevant finding was the high number of notifications with the schooling level marked as “Unknown” or “Blank”. This type of occurrence is seen as a failure to fill in the compulsory notification form of suspected or confirmed cases of violence, as it significantly impacts the precise characterization of the victims’ profile and the judicious adoption of protection and coping measures^21^.

São Paulo is the largest city in Latin America and, therefore, the characteristics of epidemiological phenomena can be different according to its regions, especially when the study object is strongly influenced by socioenvironmental factors. It is noted that the East and South regions were the ones with the highest occurrence of violence against older adults in the period outlined. These regions concentrate significant points of social vulnerability in the metropolis, with high demographic density and a large population dependent on assistance benefits^22^. Such aspects significantly potentiate risk factors for violent attitudes and perpetuation of the disease^22^ ^-^ ^24^. Other relevant evidence was the fact that the aggressions have mostly occurred in the victims’ homes during the pandemic and by close family members, especially children, and more than once. Therefore, Brazilian scholars point out that it is important for health professionals to be available to assist not only the victims, but also their offenders, as behaviors based solely on the victimized client may not be successful in interrupting the cycle of violence^25^ ^-^ ^26^.

It is interesting to point out that aged people who are married or in a stable union were not free from aggressions during the COVID-19 pandemic^27^. Psychological and/or moral violence was cited by European researchers as frequent among aged individuals^28^ ^-^ ^29^, involving the expression of verbal abuse, blackmailing, exposure to embarrassing situations, contempt and other attitudes that devalue the person being assaulted, leading to progressive psychosocial harms. Usually, the aggressors cause such actions because they know the victims’ weaknesses in depth. Thus, spouses end up being those who most hold information that can cause psychological or moral distress. The same is true in relation to sexual violence, where the aggressor is usually the aged person’s intimate partner^27^.

However, in the case of widowhood, aged individuals experienced more financial or economic violence and situations of neglect or abandonment. Absence of a partner can cause older adults to be financially exploited by younger family members and also receive little or no assistance from them. A study carried out with mental health specialists from 23 European countries mentions that these types of violence are even more complex than the others because older adults tend to naturalize the situation when they see themselves in a position of financial providers, especially in families with very low incomes and that experience unemployment^30^. In addition to that, death of the spouse exposes the aged person to neglect on the part of family members because families had often not planned to take care of that person and are now forced to do so. It is noted that, in this study, neglect and/or abandonment were also more frequent in aged people with mental disorders, showing that such diseases can trigger rejection behaviors in older adults, leading to negligence.

The confinement imposed during the COVID-19 pandemic exposed aged people to intense family life. This aspect can be one of the factors causing family stress and physical violence impulses in younger people, as found in this study. Therefore, generational conflicts were the main drivers for aggression against older adults. These conflicts are often caused by persistent differences in social, cultural and even economic values between people belonging to different age groups^21^ ^,^ ^28^ ^-^ ^30^.

As in other studies^19^ ^,^ ^27^, it was men that most perpetrated physical aggressions, mainly using physical force and/or beatings against the victims. It is noted that aggressors belonging to the age group from 25 to 59 years old have more muscle strength than aged people, potentially increasing the risk of serious bodily injury and even the chances of sequelae or complications. Physical injuries to older adults lead to potential complications to organs and tissues, increase the chances of systemic complications and can also lead to psychosocial harms associated with fear, shame and anger awakened by the aggressions^19^. In addition to that, relevant information obtained in the data analysis is that alcohol consumption was not mostly associated with the aggressions, a fact that differs from studies with other populations^19^ ^-^ ^23^.

Finally, during the service, the notifying professionals referred the older adults to services that are part of the Health and/or Social Assistance Network, according to the needs found. However, there were very few referrals of cases to the Municipal Older Adults’ Protection Council. Article 19 of the Older Adults’ Statute states that suspected or confirmed cases of violence against aged people will be object of compulsory notification by the health services, as well as that they must be referred to public security agencies^31^. In addition to that, since 2015, the city of São Paulo has a comprehensive care line for people in situations of violence that strongly recommends referral of VAOA cases to the Municipal Older Adults’ Great Protection Council, as well as it presents flows for referrals for other vulnerable populations^32^. The low number of referrals to this protection agency may imply the evaluation and monitoring of important indicators of this line of care. It is important that the professionals make adequate communication to the protection agencies for older adults as a way of guaranteeing human rights and maintaining justice.

The main study limitation is the possibility of information loss, due to late inclusion of notifications, in cases of violence still under epidemiological investigation during the current collection period, but the weakness emphasized does not make the findings unfeasible, as there is important information on the characteristics of the aggressions suffered by aged people during the COVID-19 pandemic, still scarce in the literature.

This research brings about potential contributions to Nursing and other health sciences, given that epidemiological data can support health policies for victims in a more objective way. In addition to that, it allows reflecting on measures to prevent and control the problem, as it outlines the aggressors’ profile and the characteristics of the most common types of violence. Furthermore, the findings allow planning Nursing care practices in line with the profile of victimized aged people found, as well as supporting the development of social university extension projects aimed at promoting a culture of peace, based on the reality presented.

## Conclusion

The study identified the following as the sociodemographic profile of violence against older adults in the first months of the COVID-19 pandemic: women with low schooling levels, white-skinned, aged between 60 and 64 years old and married in cases of physical, psychological or moral violence, torture and sexual violence. People aged 75 and over have mostly suffered financial or economic violence and neglect or abandonment. There was a higher occurrence of injuries in the homes and among residents of the East and South regions of the São Paulo metropolis. Thus, there were some divergences in relation to the hypotheses initially raised, evidencing particularities of violence against older adults in the pandemic context.

In relation to the aggressors, there was prevalence of males belonging to a younger age group than the victims (between 25 and 59 years old), usually with close degrees of kinship (children or spouses). Physical force/Beating was the most used type of physical violence. In turn, psychological violence was evidenced by the situations of threats. Aged people with mental disorders suffered more physical violence, while those with physical disabilities suffered more abandonment or neglect. The aggressions were perpetrated more than once, driven by generational conflicts. The referrals were predominantly to the health care and social assistance networks. However, there were few referrals to security and protection agencies, such as the Municipal Older Adults’ Protection Council.

The importance of compulsory notification of interpersonal violence against older adults during the COVID-19 pandemic is highlighted, as it allowed recognizing those who are more vulnerable, facilitating the implementation of health policies. The authors emphasize the importance of correctly filling in all the fields of the notification form, especially the victims’ schooling level.
